# Ghosts

**Published:** 1913-11

**Authors:** 


### Ghosts.

*Ghost*—My hour is almost come,
When I to sulphurous and tormenting flames,
Must render up myself.
*Hamlet*—Alas; poor ghost!
*Ghost*—Pity me not, but lend thy serious hearing
To what I shall unfold.

The ghost is ever hard to down. From the earliest times these fancy-made phantoms have pursued fearful man and scary woman. The fear of ghosts is so inborn that no amount of negative experience, no common sense, no reason or logical argument from the experience of this world, and apparently no erudition or scholastic training can down the ghost.

There is scarcely a community in this doubting and faithless land of ours, from Maine to Missouri, where there is not a haunted house, and there is certainly not a community, large or small, simple or erudite, from Massachusetts to California, that does not support one or more cults of temple building ghost-fearers or ghost-worshippers. Practically all gamblers, all bookmakers and all high financiers, as well as all burglars and safe-crackers are firm believers in the lucky and unlucky ghosts. The reading public are keen for ghost stories, and no books are more profitable to the publishers, or more called for at public libraries than books on astrology, mysticism, dream interpretation, magic, and other ghost-mongering *fachs*.

The medical profession ought to be scientific, and science ought to be agnostic of ghosts. There are, however, many physicians who believe in ghosts; that is to say, the ghost is an essential part of their philosophy of life. They are Bergsonian, even though they have never read his books.

One would naturally expect that a scientific laboratory training and that the horizon of medical study and practice would eliminate the mystical and down the ghost; but such apparently is not always the case.

In the October, 1912, number of *Science Progress*, J. S. Haldane feels called upon to expound more carefully his physiologic ghost which he first introduced to our astonished gaze in his presidential address before the Physiologic Section of the British Association. It must be admitted that, however, familiar with the vocabulary used, one is unable to understand him better than one does comprehend Mary Baker Eddy or Constantine Brunner.

Among physiologists, those who believe in ghosts are termed "vitalists" and those who do not believe in ghosts are termed "mechanists." The former ally themselves with the psychologists, the metaphysicians, the transcendental philosophers and the religionists, even with such cults as deny any physical existence at all.* The "mechanists" are naturally allied to the chemists, the physicists, the biologists and the archeologists. One would premise that the former were nowadays in a hopeless minority, but the bulk of

*The Christian Scientists and most Buddhists deny any physical existence whatever. Their position appears to them perfectly logical from a metaphysical standpoint. "The arguments of the Buddhists regarding the unreality of the objects of perception may thus be summarized. Our perceptions in dreams do not, in principle, differ from those in the waking state, and consequently the latter must be just as void and as independent of something existing beside them (their object) as the dream impressions: further examples of impressions void of really existing objects are fata morgana and mirage. As dreams or magic fata morgana are regorded as unreal by ordinary men, so this whole world is regorded by those versed in the Vedantas."—Journ. Amer. Oriental Society.

their literature would indicate that they were still in the field and that the ghost defenders were armed with entertaining arguments for which there was a sharp economic demand.

The ghost defenders and the ghost mongerers are in good company, and have many conspicuous figures as champions on the literary sward and in the pseudo-scientific bout. Notice Sir Oliver Lodge come forth in the *Contemporary Review* (October, 1912), to meet the David of the British Association. Professor Shaefer's presidential address at Dundee was not intended as a ghost-killing article, but it was of sound sense and proportionately disturbed the ghost mongerers' complacency. It was so sane and sensible that it deserves a wider reading than it has yet attained.

The Bergson cult are over active with his new ghost *élan vitale* developed in "Creative Evolution."* Like all vitalistic and dualistic philosophies it is mysterious and occult. The Society for Psychical Research, recognizing the value of Bergson's ghost, has made him their president. He and the "vitalists" among physiologists agree that material or mechanical philosophy alone cannot account for all the manifestations of life, though they admit that it does account for some and even for a large and growing number of them previously unaccountable. The ghost comes in to account for the rest. Now and then in the progress of science, some of the mysterious and occult phenomena of life presided over by the ghost become rationally and mechanistically explained and the ghost recedes. Such aggression is going on all the time. Some of the territory held for ages in the undisputed possession of the ghost gives way year by year before the assaults of scientific research and rational mechanistic philosophy.

Many, many years ago, it seems to us now, du Bois Reymond undertook to exorcise the "specter of vitalism" as he called it, which then openly held sway in physiology. For a long time he seemed to have silenced the ghost breeders, but in true occult fashion the sprite is up again, prouder than ever, clad in a garb of astonishing nomenclature borrowed, if not stolen from the ghost-slaying scientists themselves. Verbiage is one of the most conspicuous attributes of the ghost, and also the sword and shield of the ghost defenders.

This is perhaps most aptly illustrated by the new metaphysics of Hans Driesch in his "Science and Philosophy of the Organism." His is a new phase of dualism, diametrically opposite to Bergso-

*Bergson, Henri; Creative Evolution. Translated by Arthur Mitchell. New York, Henry Holt & Co., 8, p. 407, 1911.

nianism in its origin. Bergson begins in the sky and comes down to earth: Driesch begins in the ooze and rises to the heavens. We find in his "prospective potency" the embryo of his ghost, which he assures us is "a true element of nature," which shall, if we follow him aright, hereafter be known as "entelechy."

There are many manifestations of the ghost in medical literature. Let us consider for a moment one which seems to have been overlooked in America for a long time. Wilhelm Fleiss published his *Die Ablauf des Leben* in 1906, in which he brings forth a remarkable ghost, the *"family substance."* He opines the family spirit is one, though the family bodies are many, and therefore when one individual in the family is obstipated, the cathartic should not be wasted on the sick baby that accidentally manifests the malady of the "family substance" but should be administered to the parents, the grandparents, the uncles and the aunts. The sex of the family substance is marked by the mystic numbers "23" and "28." It is remarkable that this inclusive philosophy has not been taken advantage of by our aggressive "practitioners," as it would greatly enlarge their sphere of general befuddlement.

It is useless, apparently, to deny the sovereignty of the ghost over any field not already invaded by research and subjugated by rational science. Yet some authors would deny the existence of the ghost and ridicule all his pretensions. Such an one is Hugh H. Elliott,* who, in *Bedrock* (October, 1912), and *Science Progress* (January, 1913), takes the field against the very existence of any ghost, armed with biting sarcasm and cutting ridicule as well as reason. Such efforts are enlightening, amusing and wonderfully entertaining, but they fail to down the ghost.

If one were to declare that Mars is inhabited by ghosts and that in former ages they freely passed back and forth to the earth in ethereal aeroplanes, it would be impossible to refute the assertion. Strange enough after this sentence was written the writer's eyes fell upon a comment of the Editor of the Proceedings of the American Society for Psychical Research relative to Mrs. Smead's communications from Mars and Professor Flournoy's "From India to the Planet Mars." In this very coincidence the open minded might see the mechanations of the ghosts seeking to turn the "academic philistine," the writer, from his perfidious agnosticism.

The ghosts of the scientific laboratories are as invulnerable to

**See also Elliott, Hugh H. Modern Science and the illusions of Professor Bergson. London: New York, 1912. 8, p. 257.

reason and common sense as those of Lodge, Bergson, Driesch, Fleiss, Flournoy, Mrs. Smead or Hyslop. Consider those of Mach and Kirchof of the physical laboratory, Verworn of physiology and von Hanseman of pathology.

When one reads the titles not of the publishers of religious books, but in the lists of publishers of general literature or general science, and discovers the great numbers of ghost lore volumes, he begins to appreciate the extent of the ghost myth and ghost cult. The medical literature is again teeming with ghosts. They seem to be the Zeitgeist. The department of psychology and even that of psychiatry has sworn allegiance to the newest ghost. Were it not for this rebellion against the sway of science, we would have no reason to discuss the presumptuous, aggressive creature of fancy.

The time was when all diseases were in the territory of the etiologic ghost. Typhoid, diphtheria, the black death and cholera, were equally the mechanations of the evil ghost. The cruel "banshee" choked the infant with croup. The "hag" pestered the child with nightmare.

Now how changed. There is an adequate mechanistic cause for every malady known to the ancients. The typhoid, the cholera, and even the croup are in the territory subjugated by science, the insane alone are in the regency of the ghost, and the psychiatrists continue their conjuring to the neglect of rational research.—Ed. in *Lancet Clinic*, October 4, 1913.

